# Unraveling small non-coding RNAs with a significant post-transcriptional impact on breast cancer cell signaling, using a combinational sequencing approach

**DOI:** 10.1007/s10142-026-01856-6

**Published:** 2026-03-23

**Authors:** Katerina Katsaraki, Vaia K. Stafyla, Diamantis C. Sideris, Andreas Scorilas, Christos K. Kontos

**Affiliations:** 1 Department of Biochemistry and Molecular Biology, Faculty of Biology, National and Kapodistrian University of Athens, Athens, Greece; 2 Fourth Department of Surgery, University General Hospital “Attikon”, National and Kapodistrian University of Athens, Athens, Greece; 3 Institute of Applied Molecular Biology – Biomarkers and Omics Technologies, University Center of Research and Innovation “Antonis Papadakis”, National and Kapodistrian University of Athens, Athens, Greece

**Keywords:** miRNAs, tRNA-derived RNA fragments, miR-22-5p, PI3K/AKT, signaling pathway, massive parallel sequencing

## Abstract

Breast cancer (BrCa) is a predominant type of cancer with high mortality rates. The deregulation of cell signaling pathways is a hallmark of BrCa pathogenesis and disease progression. Aiming to explore novel post-transcriptional regulatory mechanisms influencing BrCa cell signaling, a combinational sequencing approach was employed by integrating small RNA sequencing with poly(A)-RNA sequencing, following the alteration of cell signaling using proteasome inhibitors. Pursuing a broad analysis of BrCa gene expression, cell lines deriving from the major molecular subtypes were incorporated. Gene expression changes were detected after treatment with both inhibitors, with the strongest overlap in the Luminal B and TNBC cells. The altered small non-coding RNA profiles suggested broad regulatory effects on genes central to BrCa biology and cell signaling. Interestingly, miR-489-3p and miR-876-3p were consistently downregulated and miR-1827 upregulated among the studied conditions, while other miRNAs were characterized with cell-line specific alterations. Several piRNAs, including piR-36,318, piR-39,245, and piR-36,036, were markedly induced. Specific tRNA-derived RNA fragments such as 5’-tRF-TRN-GTT11, 3’-tRF-TRQ-CTG3, i-tRF-TRQ-CTG3(1), and 5’-tRF-TRV-CAC14 stood out with a potential contribution to regulatory axes shaping BrCa biology. Furthermore, an upregulation of miR-22-5p/3p and a relevant downregulation of specific targets was observed in two TNBC cell lines of metastatic origin. The luciferase activity assay revealed a direct interaction of miR-22-5p with the components of the PI3K/AKT pathway, *INSR*, *ITGB8*, and *PI3KR1*. Lastly, the overexpression of mir-22 inhibited cell proliferation and migration. In conclusion, our combinational sequencing approach unraveled several small non-coding RNAs with a significant post-transcriptional impact on BrCa cell signaling.

**Supplementary Information:**

The online version contains supplementary material available at 10.1007/s10142-026-01856-6.

## Introduction

Breast cancer (BrCa) is a malignancy with a significantly high incidence and mortality rate globally. Factors contributing to BrCa pathogenesis include genetic, family, lifestyle, reproductive, and environmental factors. Individuals with mutations in high-penetrance genes such as *BRCA1*,* BRCA2*,* PTEN*,* TP53*, and *STK11* which are involved in DNA repair and cell cycle regulation, confer a substantially increased lifetime risk, with *BRCA1* and *BRCA2* pathogenic variants being associated with a 50–80% risk, and inherited in an autosomal dominant manner. Moderate-penetrance genes, including *CHEK2*, *ATM*, *PALB2*, *RAD51C*, and *RAD51D* are associated with a moderate but clinically significant increase in risk with some variants linked to specific molecular subtypes such as triple-negative BrCa. Other factors include age and personal or family history of breast or related cancers, with incidents increasing with advancing age, although tumors diagnosed in younger women often demonstrate more aggressive behavior. Molecular subtype distribution also varies by age, as luminal A tumors are more commonly observed in older women, whereas triple-negative tumors are more frequent in younger patients. Lifestyle factors such as obesity, alcohol consumption, and smoking are positively associated with risk, while regular physical activity appears to be protective. Reproductive factors that are associated with hormonal changes including age at menarche and menopause, age at first pregnancy, parity, breastfeeding duration, and oral contraceptive use influence BrCa risk and may be associated with specific molecular subtypes. Finally, environmental exposures, including contact with certain chemicals and carcinogenic agents, may further contribute to breast carcinogenesis (Hoxha et al. 2024; Lukasiewicz et al. 2021; Mao et al. 2023; Shiovitz and Korde 2015).

After many years of scientific research, different therapeutic strategies are followed based on the patient’s characteristics. Breast neoplasms are characterized based on specific markers that cancer cells carry into four major subtypes. Luminal A is described as hormone receptor-positive (HR-positive), HER2-negative, low levels of the proliferation marker Ki-67, and patients have a better prognosis in comparison to other subtypes. Luminal B is a more aggressive subtype, and it is characterized as HR-positive and HER2-negative with high Ki-67 levels or HER2-positive and low Ki-67 levels. The HER2-positive subtype is HR-negative, HER2-positive and lastly, the triple-negative (TNBC) is characterized as negative for the HR and HER2 markers. The HER2-positive subtype is more aggressive compared to Luminal B, and the TNBC subtype is more aggressive in comparison to HER2-positive as the abovementioned markers that are targeted in therapeutic strategies are missing. The majority of cancers globally are characterized as Luminal A with the second most common type being the TNBC (Tsang and Tse 2020). Despite the advancement of treatment strategies, the identification of key regulators in BrCa pathogenesis, disease progression, or treatment appears essential.

The deregulation of cell signaling pathways is a hallmark of BrCa pathogenesis and disease progression. Pathways such as the MAPK, PI3K/AKT, JAK/STAT, and other signaling pathways appear highly deregulated in BrCa cells in comparison to normal cells, as they are the mechanisms contributing to these events (Eroles et al. 2012; Ortega et al. 2020). Even though specific molecules in regulatory axes have stood out, with oncogenic or tumor-suppressive roles, current findings may appear as a drop in the ocean. Moreover, numerous contradictory results can be found in the literature.

Small non-coding RNAs (sncRNAs) are a group of untranslated < 200 nucleotides RNA molecules. This group includes microRNAs (miRNAs), transfer RNAs (tRNAs), tRNA-derived RNA fragments, small nucleolar RNAs (snoRNAs), small nuclear RNAs (snRNAs), small interfering (siRNAs), and PIWI-interacting RNAs (piRNAs). These molecules emerged having a plethora of functions participating in development and normal function as well as in pathological conditions (Diamantopoulos et al. 2018; Piao and Ma 2012). In addition to the well-established role of miRNAs in post-transcriptional gene silencing, tRNAs are essential mediators of translation, whereas tRNA derived RNA fragments have emerged as regulatory molecules capable of modulating translation, interacting with ribosomes and RNA-binding proteins, and regulating post-transcriptional gene silencing. The snoRNAs primarily guide chemical modifications of ribosomal RNAs but also contribute to alternative splicing and translational control, and snRNAs are integral components of the spliceosome. The siRNAs participate in gene regulation, chromatin organization, and transposable element repression. Similarly, piRNAs maintain genomic integrity by silencing transposable elements and modulating gene expression at both transcriptional and post-transcriptional levels. Furthermore, multiple sncRNA subclasses including miRNAs, tRNA-derived RNA fragments, and piRNAs have been extensively investigated as biomarkers and potential therapeutic targets, particularly in cancer, reflecting their central roles in gene regulatory networks (Diamantopoulos et al. 2024; Dwivedi et al. 2021; Piatek and Werner 2014; Sun et al. 2023).

A well-characterized mechanism by which some sncRNAs exert their regulatory potential is the RNA interference mechanism (Bartel 2009; Castel and Martienssen 2013). In this process, sequence-specific binding of an RNA suppresses gene expression after the incorporation of RNA regulators with Argonaute proteins in the RNA-induced silencing complex (RISC). The miRNAs have emerged as key factors in this process where they may reduce the bioavailability of a complementary mRNA target, leading to its degradation or its translational repression. A similar function has been highlighted for other RNA molecules such as tRNA-derived RNA fragments and piRNAs (Castel and Martienssen 2013; Xie et al. 2020).

Multifarious functions of sncRNAs have been described in relation to BrCa affecting a plethora of processes contributing to carcinogenesis or disease progression. The miRNAs is the most studied class with specific miRNA-mRNA relations emerging as significant regulators of cancer cell cellular processes contributing to cell viability, proliferation, differentiation, and tumor metastasis (Maryam et al. 2021). Studies uncovering the role of tRNA-derived RNA fragments and piRNAs have also gradually emerged in the last few years, proposing a similar miRNA-like role. Moreover, snRNAs and snoRNAs appear to also have specific functions in BrCa as both mRNA maturation and translation are deregulated (Dvorska et al. 2021). In the context of BrCa, information about the regulatory potential of sncRNAs, with the exception of miRNAs, is still limited. In BrCa cell signaling, specific miRNAs appear to affect signaling pathways, including MAPK, PI3K/AKT, NFκB, Wnt/β-catenin and Notch, by targeting specific mRNA targets and leading to the deregulation of normal cellular processes (Abolghasemi et al. 2020; Papatsirou et al. 2021).

Aiming to explore novel post-transcriptional regulatory mechanisms influencing BrCa cell signaling, we performed a combinational sequencing approach in BrCa cell lines representing different molecular subtypes. Cells were treated with proteasome inhibitors (PIs) in order to trigger cellular signaling pathways and investigate the regulation at a post-transcriptional level. PIs inhibit the physiological proteolytic process leading to apoptosis (Nunes and Annunziata 2017). Given the incorporation of PIs in BrCa clinical trials (Han et al. 2023), the study aims to provide insights into the sncRNA regulatory potential on BrCa cell signaling.

## Methodology

### Cell culture conditions and treatments

Six BrCa cell lines belonging to different molecular subtypes were cultured at a confluency of 60–80% in a humidified incubator at 37℃ and 5% CO_2_. The Luminal A MCF-7, Luminal B BT-474, HER2-positive SK-BR-3, and the TNBC MDA-MB-468, MDA-MB-231, and BT-20 cell lines we cultured according to the ATCC guidelines. Standard curves and proliferation curves were constructed in order to treat cells at a similar proliferation rate. Apoptosis was assessed by the Caspase-3 activity colorimetric assay, following the manufacturer’s instructions (Elabscience, Houston, TX, USA), and the determination of the IC_50_ values of treatment of cells with bortezomib and carfilzomib was performed, as previously described (Katsaraki et al. 2024). At 24 h post-treatment, DNA and RNA were extracted using the TRItidy G reagent (AppliChem GmbH, Darmstadt, Germany). Their quantity, quality, and integrity were assessed by combining fluorometry with the Qubit system, spectrophotometry by calculating the 260/280 ratio, and automated electrophoresis using the 4150 TapeStation System (Agilent Technologies, Winooski, VT, USA).

### Small RNA sequencing

Small RNA sequencing libraries were prepared from 700 ng of total RNA, using the MGIEasy Small RNA Library Prep Kit by MGI-Tech (Shenzhen, China). Total RNA extracted from the MCF-7, BT-474, SK-BR-3, and MDA-MB-468 cell lines was used. Size selection of libraries was performed using the Pippin-Prep system and pre-cast 3% agarose gel cassettes (Sage Science, Beverly, MA, USA), followed by combining them in equimolar amounts for sequencing. The quality and quantity of libraries and pool were performed using the High Sensitivity D1000 DNA ScreenTape and the 4150 TapeStation System (Agilent Technologies). Sequencing was performed on a DNBSEQ-G400 (MGI-Tech) with a SE50 read sequencing approach.

### Poly(A)-RNA sequencing

For the construction of poly(A)-RNA libraries poly(A) RNA was isolated from 1 µg of total RNA from the abovementioned treated cell lines. The selection was performed in the same RNA samples that were used in the small RNA sequencing using the NEBNext Poly(A) mRNA Magnetic Isolation Module (New England Biolabs Ltd., Hitchin, UK), according to the manufacturer’s instructions. Selection was assessed using the High Sensitivity RNA, and the 4150 TapeStation System (Agilent Technologies). Libraries were prepared using the MGIEasy RNA Directional Library Prep Set (MGI-Tech), combined equimolarly, and the pool was sequenced with a DNBSEQ-G50 (MGI-Tech) sequencer following a PE100 read sequencing approach.

### Bioinformatic analysis of sequencing results

Publicly available algorithms as well as the Partek Flow Genomic Analysis Software (Illumina, San Diego, CA, USA) were used for the analysis of sequencing reads. For the analysis of the small RNA sequencing reads, reads were aligned with the Bowtie algorithm. The poly(A)-RNA sequencing reads were aligned using the STAR aligner to the GRCh38 (hg38) (Dobin et al. 2013; Langmead et al. 2009). Reads were normalized by Counts Per Million. Annotation was performed using data from RefSeq (O’Leary et al. 2016), miRBase (Kozomara et al. 2019), DASHR (Kuksa et al. 2019), and MINTbase (Pliatsika et al. 2016), for the identification of different subtypes of RNAs. Gene Specific Analysis followed for the identification of differentially expressed genes after treatment with the inhibitors. Fold changes ≤ 1/2 or ≥ 2, were considered significant. In the last step, Gene Set Enrichment Analysis was performed for the identification of pathways affected after the treatments, according to the Kyoto Encyclopedia of Genes and Genomes (KEGG), as well as biological processes and molecular functions according to Gene Ontology (GO). Pathway enrichment analysis plots were constructed in RStudio.

### Bioinformatic analysis for the integration of the sequencing results

Target prediction for tRNA-derived RNA fragments and miRNAs with significant differences in their levels was performed. Databases and target prediction algorithms including TargetScanHuman v.8 (McGeary et al. 2019), miRDB v.6 (Chen and Wang 2020), and miRDIP v.5 (Hauschild et al. 2023), were used. Aiming to identify regulatory relations in specific signaling pathways, we limited our analysis to genes with a significant difference that participate in the MAPK, PI3K/AKT, and the NFκB pathways, according to KEGG. Experimentally validated miRNA-mRNA binding relations were identified using the miRTarBase v.9. (Huang et al. 2022), and TarBase v.9 databases (Skoufos et al. 2024), and tRNA-derived RNA fragment-mRNA with tRFTar (Zhou et al. 2021), and tRFUniverse (La Ferlita et al. 2024). Furthermore, an extensive review of literature revealed regulatory miRNA-mRNA combinations in other malignant conditions.

### Identification of significant differences in gene expression using real-time polymerase chain reaction

Aiming to validate the sequencing results, we quantified the levels of selected miRNAs and mRNAs 24 h post-treatment. In this step, samples from the MDA-MB-231 and BT-20 cell lines, belonging to the TNBC molecular subtype were also included. For the levels of miRNAs, polyadenylation of 400 ng of total RNA was performed using E. *coli* poly(Α) RNA polymerase (New England Biolabs Ltd) followed by a reverse transcription reaction using an oligo-dT adaptor primer and M-MLV reverse transcriptase (Invitrogen, Thermo Fisher Scientific, Waltham, MA, USA). For the levels of mRNAs, 400 ng of total RNA was reverse transcribed using an oligo-dT primer. Specific primers were designed for the amplification of targets (Table S1) and a universal reverse primer for the amplification of miRNAs. Lastly, qPCR reactions were performed using a 1:4 diluted cDNA and KAPA SYBR FAST qPCR master mix (2X) (Kapa Biosystems Inc., Woburn, MA, USA), in a 10 µL final volume, using a QuantStudio 5 system (Applied Biosystems, Waltham, MA, USA). Relative quantification was performed using the 2^−ΔΔCt^ method, *GAPDH*, and the average of *SNORD43* and *SNORD44* as reference genes (Livak and Schmittgen 2001). Fold changes ≤ 1/2 or ≥ 2 were considered significant.

### Construction of plasmids overexpressing mir-22 and 3´ UTRs of its predicted targets

From the aforementioned workflow, miR-22 emerged as a promising molecule to further elucidate. mir-22 as well as upstream and downstream regulatory regions were cloned into the pCMV6-Neo vector (OriGene Technologies Inc., Rockville, MD, USA). For this purpose, a PCR reaction for the amplification of the selected region was performed using specific primers (Table S1), DNA from the MDA-MB-468 cell line, and KAPA Hi-Fi Hotstart Ready mix (Kapa Biosystems Inc.). A hybridization temperature of 65℃ for 15 s and amplification for 15 s was performed for 30 cycles. The specificity of mir-22 regions was assessed by electrophoresis and Sanger sequencing. The PCMV6-Neo vector and the amplified mir-22 region were digested using the SacI-HF and EcoRI-HF restriction enzymes, followed by ligation of the two nucleic acids using the T4 ligase and the appropriate buffer.

For the construction of plasmids overexpressing 3´ UTRs of its predicted targets, sequences were synthesized by Eurofins Genomics and ligated by performing Gibson assembly into the psiCHECΚ-2 (Promega, Madison, WI, USA) Firefly and Renilla luciferase-expressing vector. The vector was previously digested using the XhoI and NotI-HF restriction enzymes and the ligation was performed using the NEBuilder HiFi DNA Assembly Master Mix in a final volume of 20 µL, according to the manufacturer’s instructions. The sequences of the 3´ UTR of *INSR*, *ITGB8*, *MRAS*, *MYB*, *PIK3R1*, *PRLR*, and *RBL2* are presented as Supplementary material. Lastly, it is important to mention that *MRAS* was the only miR-22-3p predicted target. Temperatures and concentrations used were according to the manufacturer’s instructions and all material was from New England Biolabs Inc., if not specified. Lastly, all reactions were performed in a Veriti 96-Well Thermal Cycler (Applied Biosystems).

Bacterial DH5a cells were transformed using each constructed plasmid followed by a selection based on ampicillin resistance, and liquid cell culture. Plasmids were isolated using specific columns (Macherey-Nagel GmbH & Co. KG, Düren, Germany). Lastly, the constructs were assessed by performing PCR with specific primers for the multiple cloning site of each vector (Table S1).

### Overexpression of mir-22 and quantification of its targets in MDA-MB-468 cells

The pCMV6-Neo-mir-22 and the empty pCMV6-Neo plasmid were used for the transfection of the MDA-MB-468 TNBC cell line. Specifically, 300,000 cells per well were seeded in 6 wells and transfection was performed after 24 h using 2 µg from the pCMV6-Neo-mir-22 plasmid or the empty vector, and the jetPRIME transfection reagent (Polyplus, Illkirch, France). Transfection reagents were also used in a mock reaction. RNA was isolated 24 h post-transfection and was used for the quantification of miRNAs and mRNA transcripts with real-time qPCR, as mentioned above.

### Assessment of the cellular effects of mir-22 overexpression

Following the abovementioned transfection, a morphological observation in an Axio Vert.A1 (Carl Zeiss, Oberkochen, Germany) inverted microscope, as well as an assessment of cellular proliferation, and a wound healing assay were performed. Cellular proliferation was assessed using the Sulforhodamine B assay (Sigma-Aldrich, St. Louis, MO, USA) and the results of the wound healing assay were assessed using the ImageJ program.

### Luciferase reporter assays involving miR-22-5p/3p and their selected potential targets

In the next step, 20,000 cells per well of the MDA-MB-468 cell line were seeded in 96 well plates and a co-transfection of 50ng of pCMV6-Neo-mir-22 or the empty vector and 50 ng from the psiCHECK-2-3´UTR was performed. Moreover, wells with co-transfection of empty vectors and wells including only transfection reagents were also used as controls. Lastly, the luciferase activity assay was performed using the Dual-Luciferase^®^ Reporter Assay System (Promega Corporation) and the microplate spectrophotometer Spark (Tecan, Männedorf, Switzerland).

## Results

### Differential expression of genes after treatment of BrCa cell lines with PIs

**Fig. 1:**
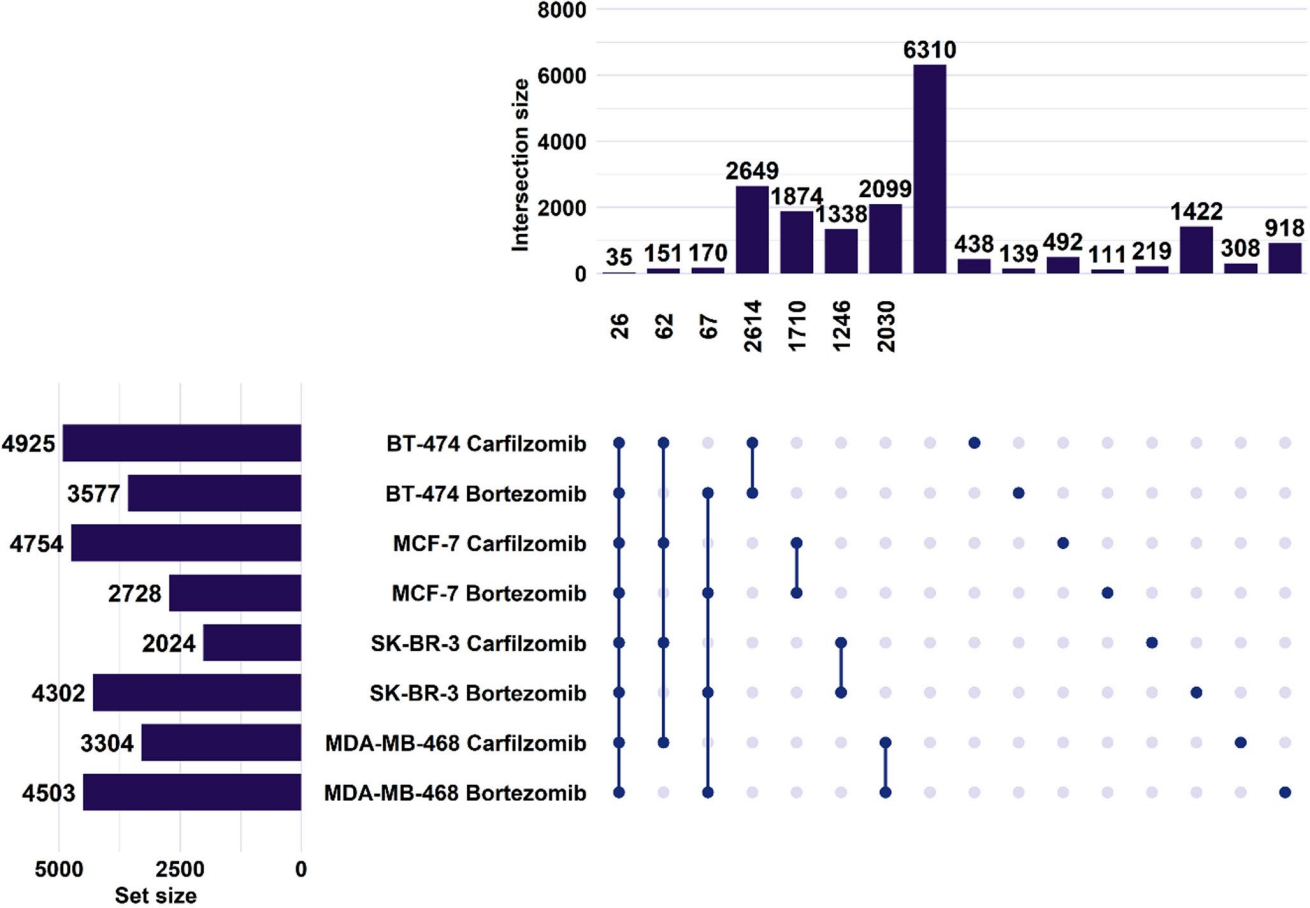
Differentially expressed mRNAs across BrCa cell lines following treatment with PIs. Horizontal bars (left) show the total number of DEGs per condition (set size). Connected dots indicate intersection of conditions. Vertical bars (top) represent the number of DEGs in each intersection (intersection size). Numbers below the vertical bars indicate genes with consistent directionality of expression change either upregulation or downregulation within that intersection. Numbers above single dots correspond to genes uniquely altered in each specific condition

The poly(A)-RNA sequencing approach revealed a considerable number of genes with altered expression after treatment with the PIs. As depicted in Fig. 1, the most differentially expressed genes (DEGs) may be observed in the BT-474 cell line after treatment with carfilzomib (4,925 genes), followed by the MCF-7 cell line (4,754 genes). On the contrary, in the HER2-positive (4,302 genes) and the TNBC cell lines (4,503 genes), bortezomib appears more effective at a transcriptomic level than carfilzomib, as more genes are differentially expressed. Furthermore, the greatest overlap in DEGs, regardless of whether they show concordant or opposing regulation between PIs, can be observed in the Luminal B (2,649 genes) and TNBC (2,099 genes) cell lines.

**Fig. 2:**
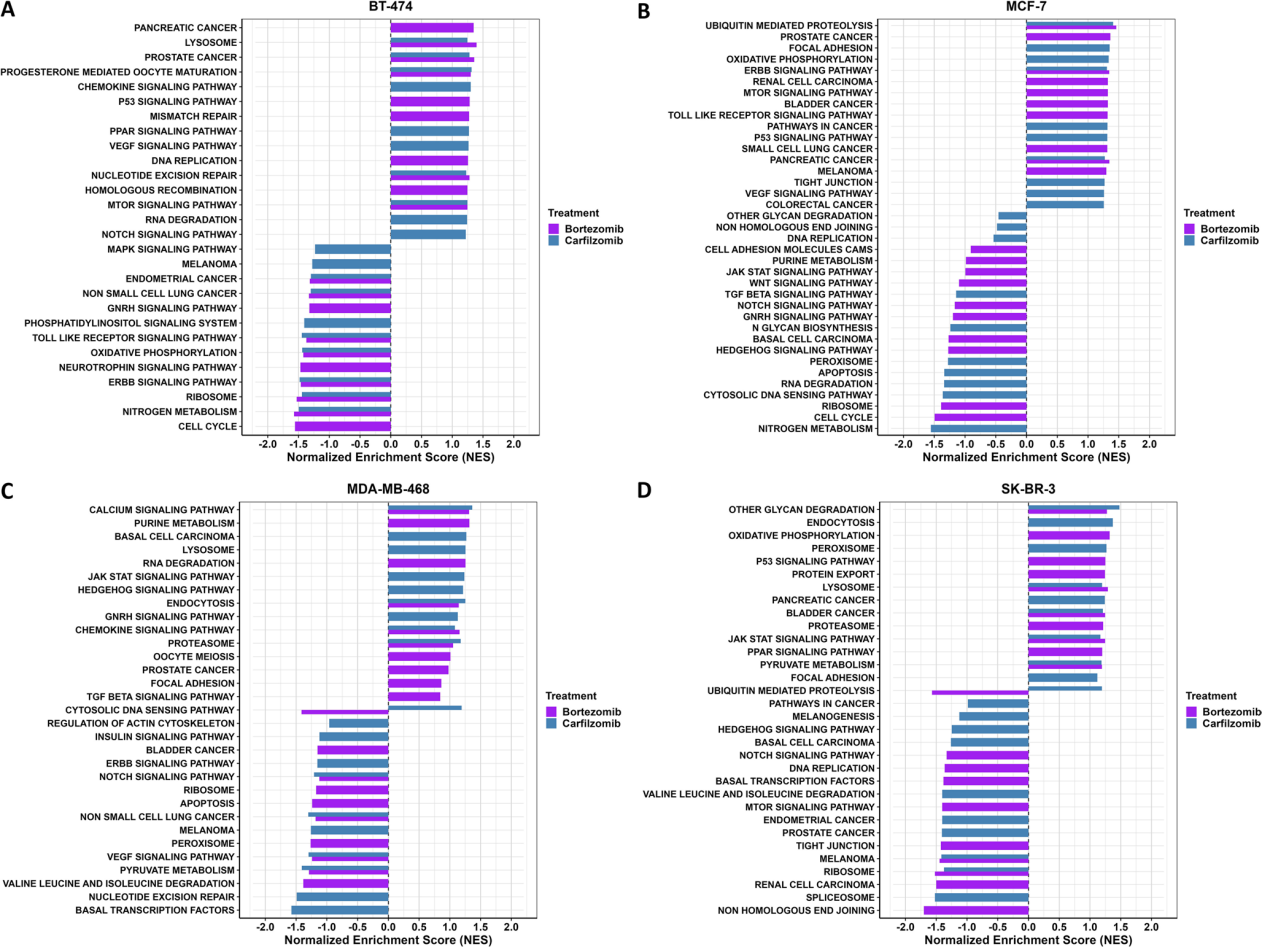
Enriched KEGG pathways based on poly(A)-RNA sequencing results, after treatment of the BT-474 **(A)**, MCF-7 **(B)**, MDA-MB-468 **(C)**, and SK-BR-3 **(D)** cell lines with bortezomib and carfilzomib

The same appears to be observed for concordant regulation of DEGs between PIs, with 2,614 DEGs in Luminal B and 2,030 in TNBC cell lines. Twenty-six genes, in a total of thirty-five with significant altered expression, displayed consistent upregulation or downregulation across all treatments. Additionally, 62 genes in all samples treated with carfilzomib, and 67 genes in all samples treated with bortezomib showed consistent directional changes. Furthermore, a large subset of genes (1,422) was uniquely deregulated in SK-BR-3 cells after bortezomib treatment, highlighting potential cell line–specific effects. Gene set enrichment analysis (Fig. 2) indicated significant enrichment of genes associated with oncogenic pathways, such as MAPK and PI3K/AKT signaling, in all four BrCa cell lines, while to variable degrees activating significant cellular processes. For example, in the MDA-MB-468 cell line significant protein related processes are affected such as the proteasome, lysosome, and ribosome, along with notable changes in the PI3K/AKT pathway, whereas prominent alterations across regulatory axes in other types of malignancies may be observed in the BT-474 and SK-BR-3 cell lines.

In the next step, we focused only on genes that are part of the MAPK, PI3K/AKT, and NFκB signaling pathways (Table S2). The most genes contributing to cell signaling and with altered expression were observed in the MDA-MB-468 cell line. Interestingly, *EGF*, *GADD45A*, *GADD45G*, *HSPA1B*, and *SPP1* appeared significantly upregulated in seven conditions studied, after treatment with PI. Furthermore, *CXCL2*,* HSPA6*, and *LY96* appear upregulated in the two luminal and the TNBC cell lines after treatment with both the PIs. The *FGFR3*, and *MAP3K12* appeared downregulated, and *PPP2R2C* upregulated in the BT-474 and MDA-MB-468 cell lines. Moreover, the expression of numerous genes appeared altered after treatment with both inhibitors, in specific cell lines such as *CDKN1A*, *INSR*, *MRAS*, *PIK3R1*, and *PRLR* in the MDA-MB-468 cell line.

### Differential expression of sncRNAs after treatment of BrCa cell lines with PIs

**Fig. 3:**
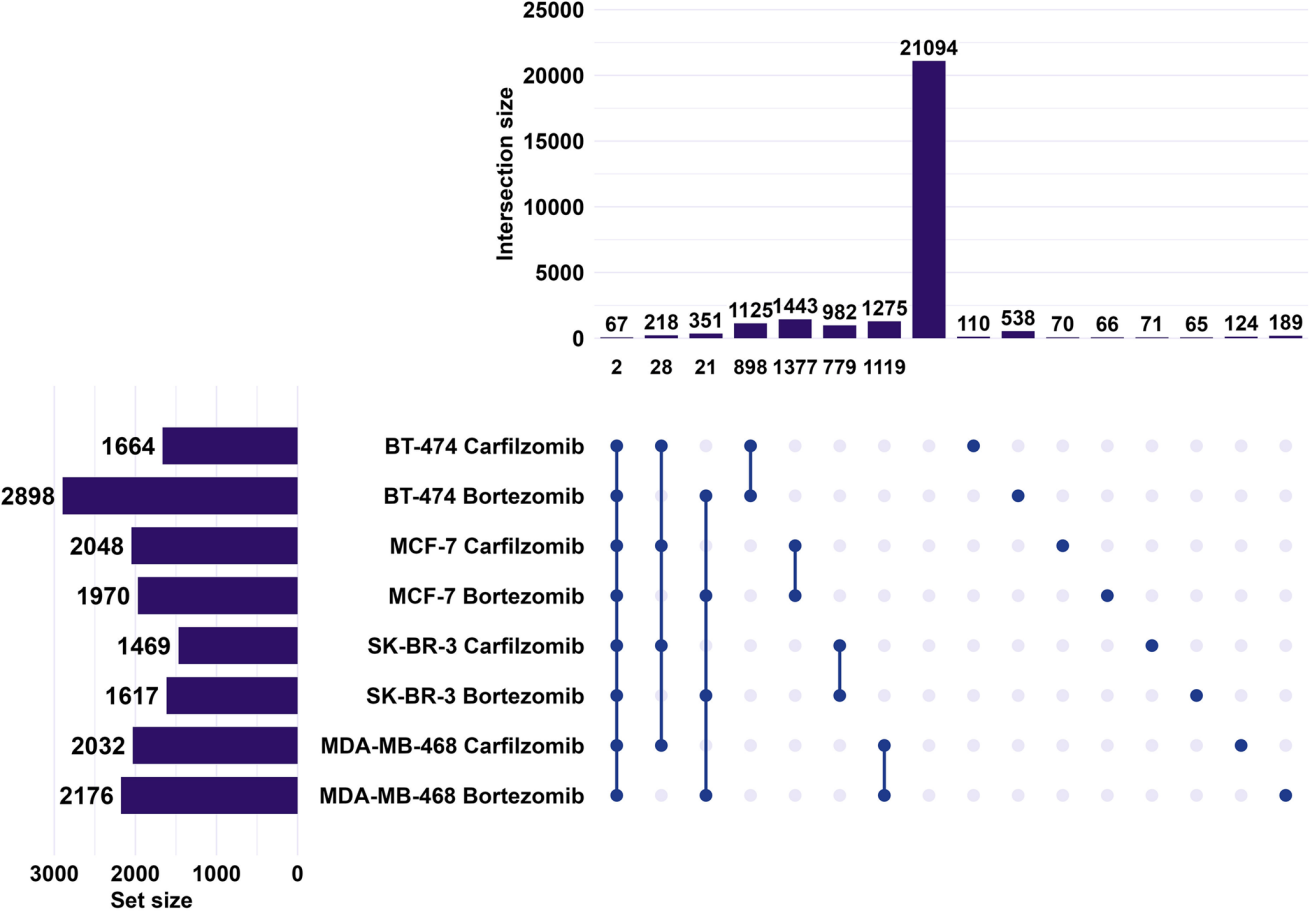
Differentially expressed sncRNAs (except for miRNAs) across BrCa cell lines following treatment with PIs. Horizontal bars (left) show the total number of sncRNAs per condition (set size). Connected dots indicate intersection of conditions. Vertical bars (top) represent the number of sncRNAs in each intersection (intersection size). Numbers below the vertical bars indicate sncRNAs with consistent directionality of expression change either upregulation or downregulation within that intersection. Numbers above single dots correspond to sncRNAs uniquely altered in each specific condition

The differential expression of sncRNAs unraveled similarities and differences between the conditions studied. Only two tRNA-derived RNA fragments from the cohort of sncRNAs (except for miRNAs) appeared consistently regulated among the 67 sncRNAs that were significantly altered across all eight cases (Fig. 3A), highlighting a limited number of universally and directionally concordant sncRNA alterations. Overall, the number of significantly altered sncRNAs per condition ranged with the lower number being observed in the SK-BR-3 cell line after treatment with carfilzomib and the highest in BT-474 after treatment with bortezomib. Furthermore, a comparable number of sncRNAs was concordantly altered by both PIs within each cell line, ranging from 779 in SK-BR-3 to 1,377 in MCF-7 cells. Notably, over 500 sncRNAs excluding the miRNAs category were significantly altered in the BT-474 cell line after treatment with bortezomib uniquely.

From this analysis, molecules such as piR-36,318 in BT-474, piR-39,245 in MCF-7, and piR-36,036 in SK-BR-3, appear as the most significantly upregulated piRNAs after treatment with both PIs. In parallel, the levels of several tRNA-derived RNA fragments are similar across treatment with both drugs, such as i-tRF-TRE-CTC12 in SK-BR-3, and i-tRF-TRD-GTC4(2) in MCF-7. A more distinct profile is observed in the most significantly altered miRNAs (Table 1). In addition, the levels of miRNAs, tRNA-derived RNA fragments, piRNAs, snRNAs, snoRNAs, and Y RNAs appeared to be affected with the majority of transcripts being tRNA-derived RNA fragments or miRNAs.

**Table 1:** tRNA-derived RNA fragments, miRNAs, and piRNAs with the greatest alterations in expression for each cell line and inhibitor combination

Cell line	Proteasome inhibitor	sncRNA	Regulation
BT-474	Carfilzomib	leader-NMTRL-TAA3-1, miR-4717-5p, piR-36,318	Upregulation
	Bortezomib	i-tRF-TRQ-CTG3(1), miR-4492, piR-36,318	
MCF-7	Carfilzomib	5’-tRF-TRN-ATT1, miR-3197, piR-39,245	
	Bortezomib	trailer-TRQ-CTG3-1, miR-199b-5p, piR-39,245/piR-48,252	
MDA-MB-468	Carfilzomib	trailer-TRG-GCC4-1, miR-642a-3p, piR-40,494	
	Bortezomib	i-tRF-TRL-AAG2(3), miR-4306, piR-44,397	
SK-BR-3	Carfilzomib	i-tRF-TRE-CTC12, miR-4306, piR-36,036	
	Bortezomib	i-tRF-TRE-CTC12, miR-320e, piR-36,036	
BT-474	Carfilzomib	i-tRF-TRL-CAG1(5), miR-6741-5p, piR-39,592	Downregulation
	Bortezomib	trailer-TRG-GCC4-1, miR-3143, piR-39,592	
MCF-7	Carfilzomib	i-tRF-TRD-GTC4(2), miR-7109-5p, piR-43,246	
	Bortezomib	i-tRF-TRD-GTC4(2), miR-4501, piR-47,274	
MDA-MB-468	Carfilzomib	i-tRF-TRQ-CTG3(2), miR-663a, piR-37,124	
	Bortezomib	5’-tRF-TRG-CCC4, miR-6831-5p, piR-53,119	
SK-BR-3	Carfilzomib	5’-tRF-TRN-GTT8, miR-301b-5p, piR-37,124	
	Bortezomib	i-tRF-TRY-GTA1(9), miR-4508, piR-48,419/piR-50,602	

### tRNA-derived RNA fragments with significant regulatory potential in substantial cellular processes and signaling pathways

Differential expression analysis identified multiple tRNA-derived RNA fragments significantly altered across the BrCa cell lines following treatment with PIs. Among these, 5’-tRF-TRN-GTT11 (Asn^GTT^), and 3’-tRF-TRQ-CTG3 (Gln^CTG^) appeared significantly downregulated in all the combinations of PI and cell line studied. In contrast, i-tRF-TRK-TTT14(1) (Lys^TTT^), and i-tRF-TRQ-CTG3(1) (Gln^CTG^) are two of the most significantly upregulated fragments after treatment with both inhibitors in the BT-474 cell line, Leader-NMTRQ-TTG1-1 (Gln^TTG^) in the MCF-7, and 5’-tRF-TRV-CAC14 (Val^CAC^), 5’-tRF-NMTRL-TAA1 (Leu^TAA^), and i-tRF-TRI-TAT3(1) (Ile^TAT^) in the MDA-MB-468. On the contrary, the levels of 5’-tRF-TRN-GTT8 (Asn^GTT^) are significantly attenuated in the SK-BR-3 cell line.

To investigate the regulatory potential of the most significantly altered tRNA-derived RNA fragments, target prediction and CLIP-seq-supported interaction analyses were performed. 5’-tRF-TRN-GTT11 appears to regulate *CCND1*, *FLNC*, *LAMC2*, and *PTGS2*, based on CLIP-seq data analysis. Furthermore, i-tRF-TRK-TTT14(1) appears to regulate *CACNB2*, and *PLA2G4C*, whereas i-tRF-TRQ-CTG3(1) is predicted to target *BDNF*, *CXCL12*, *EFNA5*, and *MEF2C*. In addition, 5’-tRF-TRV-CAC14 is predicted to target *CACNA2D2*, *LAMC1*, *MTOR*, *PIK3CG*, and *PRKACB*, while 5’-tRF-NMTRL-TAA1 is predicted to regulate *ANGPT2*, *CACNB4*, *CD14*, *CDK6*, *DUSP3*, *GNB4*, *IL2*, *IL6R*, *ITGB8*, *MDM2*, *PIK3CG*, *PPP3CB*, *PRKAA2*, *RAP1B*, *TGFBR1*, and *VEGFA*. Further analyzing the regulatory capacity of the most significantly altered tRNA-derived RNA fragments based on the validated and predicted gene targets, they appear to affect several cellular pathways, including the MAPK, PI3K/AKT, and NFκB signaling pathways (Table 2 and Table S3). Furthermore, they appear to influence BrCa biology by targeting several highly significant genes, including *CDH1*, *MYC*, *RB1*, *ZEB1*, *TWIST1*, and *VEGFA*.

### Identification of miRNAs with altered expression and elucidation of the related enriched pathways

**Fig. 4:**
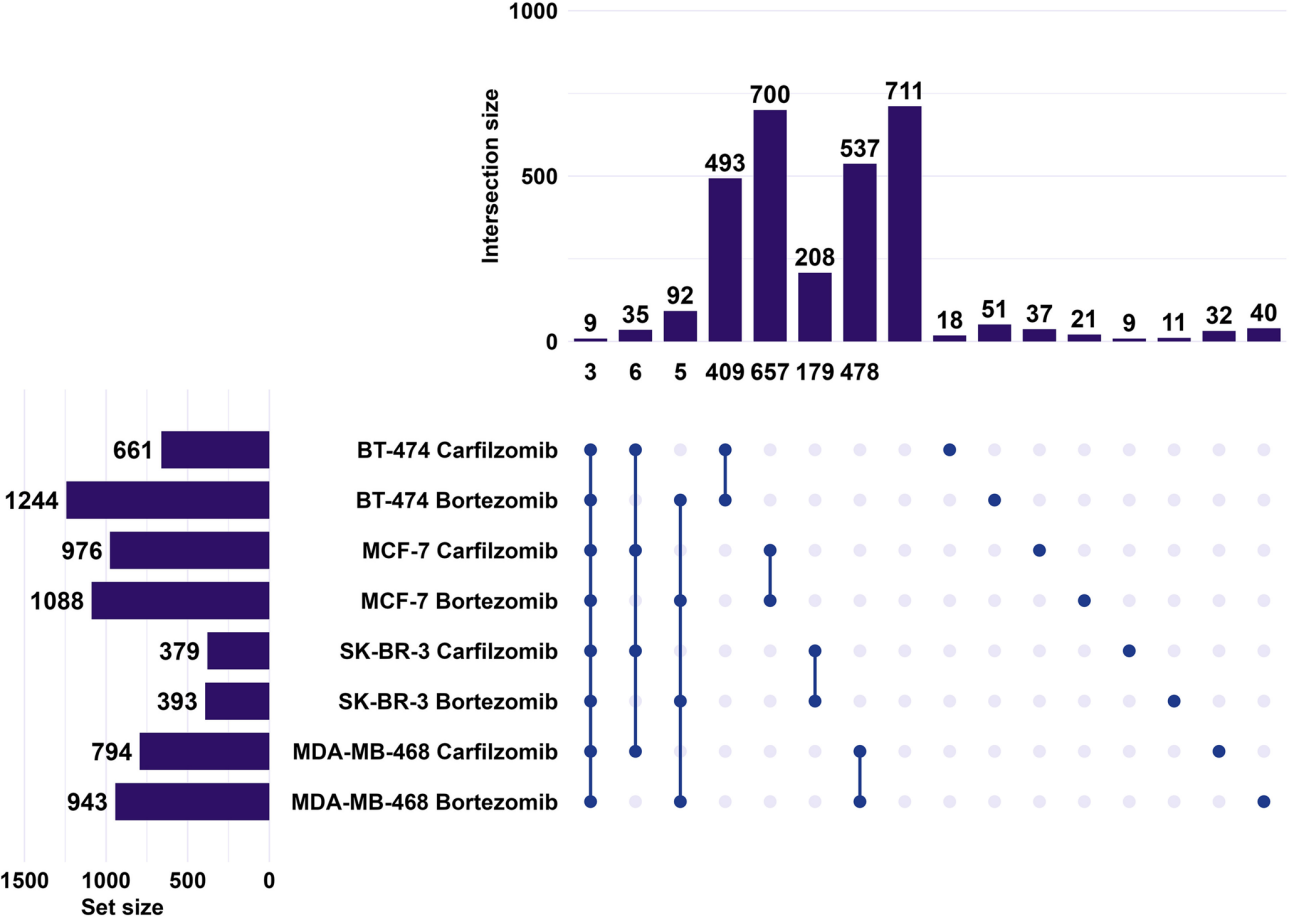
Differentially expressed miRNAs across BrCa cell lines following treatment with PIs. Horizontal bars (left) show the total number of miRNAs per condition (set size). Connected dots indicate intersection of conditions. Vertical bars (top) represent the number of miRNAs in each intersection (intersection size). Numbers below the vertical bars indicate miRNAs with consistent directionality of expression change either upregulation or downregulation within that intersection. Numbers above single dots correspond to miRNAs uniquely altered in each specific condition

In the next step, the miRNAs with a significant alteration in their expression following treatment in each combination were further analyzed (Fig. 4 and Table S4). Interestingly, miR-489-3p and miR-876-3p appeared consistently downregulated, and miR-1827 upregulated in all eight conditions studied. The total number of significantly altered miRNAs ranged from 379 in SK-BR-3 cells treated with carfilzomib to 1,244 in BT-474 cells treated with bortezomib. In all four cell lines, bortezomib resulted in a higher number of altered miRNAs compared to carfilzomib, with the greatest difference observed in the BT-474 cell line. Only six miRNAs appeared with similar upregulation or downregulation out of thirty-five miRNAs that are significantly altered in samples treated with carfilzomib. For bortezomib treated samples, only five miRNAs out of ninety-two with significant alteration appeared with concordant alteration in their levels. Lastly, the greatest overlap of miRNAs altered by both inhibitors within the same cell line was observed in MCF-7 cell line (657), followed by MDA-MB-468 (478), BT-474 (409), and SK-BR-3 (179), indicating variable concordance between inhibitors across molecular subtypes. Among the shared miRNAs, miR-2276-5p was prominently upregulated in MCF-7 cells following treatment with both inhibitors, and miR-320e in the SK-BR-3 cells. Overall, the majority of differentially expressed miRNAs were specific to individual cell lines and treatment conditions, with limited overlap across all eight datasets (Table S5).

**Table 2:** Characteristics and regulatory potential of tRNA-derived RNA fragments with the greatest alterations in expression in each cell line and inhibitor combination

Cell line	Inhibitor	tRNA-derived RNA fragment	Expression	Type of fragment	Amino acid and anticodon of parental tRNA	Targets^1^ based on CLIP-seq	miRDB targets^2^	Affected pathways
BT-474	Carfilzomib	Leader-NMTRL-TAA3-1	Upregulated	Leader	Leu^TAA^			
	Bortezomib	i-tRF-TRQ-CTG3(1)		i-tRF	Gln^CTG^		*BDNF*,* CXCL12*,* EFNA5*,* MEF2C*	MAPK, PI3K/AKT, NFκB
MCF-7	Carfilzomib	5’-tRF-TRN-ATT1		5’-tRF	Asn^ATT^		*FAS*,* FOXO3*,* GDNF*,* ITGA8*,* NRAS*,* PHLPP1*,* PIK3CA*,* PIK3R1*,* PPP2R1A*	MAPK, PI3K/AKT
	Bortezomib	Trailer-TRQ-CTG3-1		Trailer	Gln^CTG^			
MDA-MB-468	Carfilzomib	Trailer-TRG-GCC4-1			Gly^GCC^			
	Bortezomib	i-tRF-TRL-AAG2(3)		i-tRF	Leu^AAG/TAG^			
SK-BR-3	Carfilzomib	i-tRF-TRE-CTC12			Glu^CTC^		*IRAK4*,* LTBR*,* MAP4K3*,* MRAS*,* RPS6KB1*,* SOS1*	MAPK, PI3K/AKT, NFκB
	Bortezomib							
BT-474	Carfilzomib	i-tRF-TRL-CAG1(5)	Downregulated	i-tRF	Leu^CAG^		*IL1RAP*,* MAP3K5*,* PDGFA*,* YWHAB*	MAPK, PI3K/AKT
	Bortezomib	Trailer-TRG-GCC4-1		Trailer	Gly^GCC^			
MCF-7	Carfilzomib	i-tRF-TRD-GTC4(2)		i-tRF	Asp^GTC^	*BCL2L1*,* CDC25B*,* CDK6*,* FGFR1*,* LAMA5*,* PLAU*,* YWHAE*	*CRKL*,* FASLG*,* PPM1A*	MAPK, PI3K/AKT, NFκB
	Bortezomib							
MDA-MB-468	Carfilzomib	i-tRF-TRQ-CTG3(2)			Gln^CTG^		*GSK3B*,* PLA2G4A*,* PPP2R5D*	MAPK, PI3K/AKT
	Bortezomib	5’-tRF-TRG-CCC4		5’-tRF	Gly^CCC^			
SK-BR-3	Carfilzomib	5’-tRF-TRN-GTT8			Asn^GTT^		*FAS*,* FOXO3*,* GDNF*,* ITGA8*,* NRAS*,* PHLPP1*,* PIK3CA*,* PIK3R1*,* PPP2R1A*	MAPK, PI3K/AKT
	Bortezomib	i-tRF-TRY-GTA1(9)		i-tRF	Tyr^GTA^		*COL4A2*,* IGF1*,* MAP2K4*,* MAP3K1*,* MAPK13*,* PIK3R3*,* PRKACB*,* YWHAG*	MAPK, PI3K/AKT

^1^Only targets contributing to cell signaling pathways are illustrated.

^2^Only targets with a prediction score of ≥ 80% that also contribute to cell signaling pathways are illustrated.

**Fig. 5:**
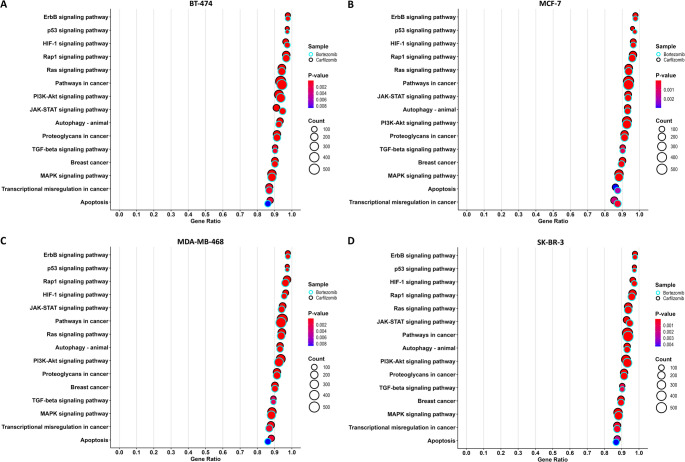
Selected enriched KEGG pathways affected by the two inhibitors, based on small RNA sequencing, in the BT-474 **(A)**, MCF-7 **(B)**, MDA-MB-468 **(C)**, and SK-BR-3 **(D)** cell lines

Based on the predicted targets, the regulatory effect that occurs in pathways for every combination is presented in Fig. 5. Significant pathways such as pathways in cancer, BrCa, apoptosis, p53 signaling, as well as the MAPK and PI3K/AKT, were enriched based on predicted miRNA targets, suggesting a potential impact of altered miRNAs on cell signaling networks. In addition, important biological processes including regulation of gene expression at multiple levels, regulation of translation, cell-cycle control, and proteasome-mediated protein catabolism seem to be affected. Aiming to investigate regulatory axes contributing to cellular signaling pathways, we focused on genes that participate in specific signaling pathways. At the same time, we focused on predicted interactions where a miRNA is upregulated, and its mRNA target is downregulated after treatment of a cell line with an inhibitor. In addition, already confirmed interactions in BrCa, interactions in other cancer types, and potential interactions that were worth further studying were considered.

### Validation of differential expression of selected miRNAs and genes

Part of the sequencing results were also confirmed experimentally, using the relative quantification method. Quantification was performed using the relative 2^−ΔΔCt^ quantification method, after checking any limitations. From the extended above-mentioned analysis, miR-22-5p/3p appeared as the most promising for further analysis. Based on the predicted targets of each miRNA, the possibility of miRNA expression, and clinical trials, the overexpression of mir-22 in TNBC was chosen for further analysis.

**Fig. 6:**
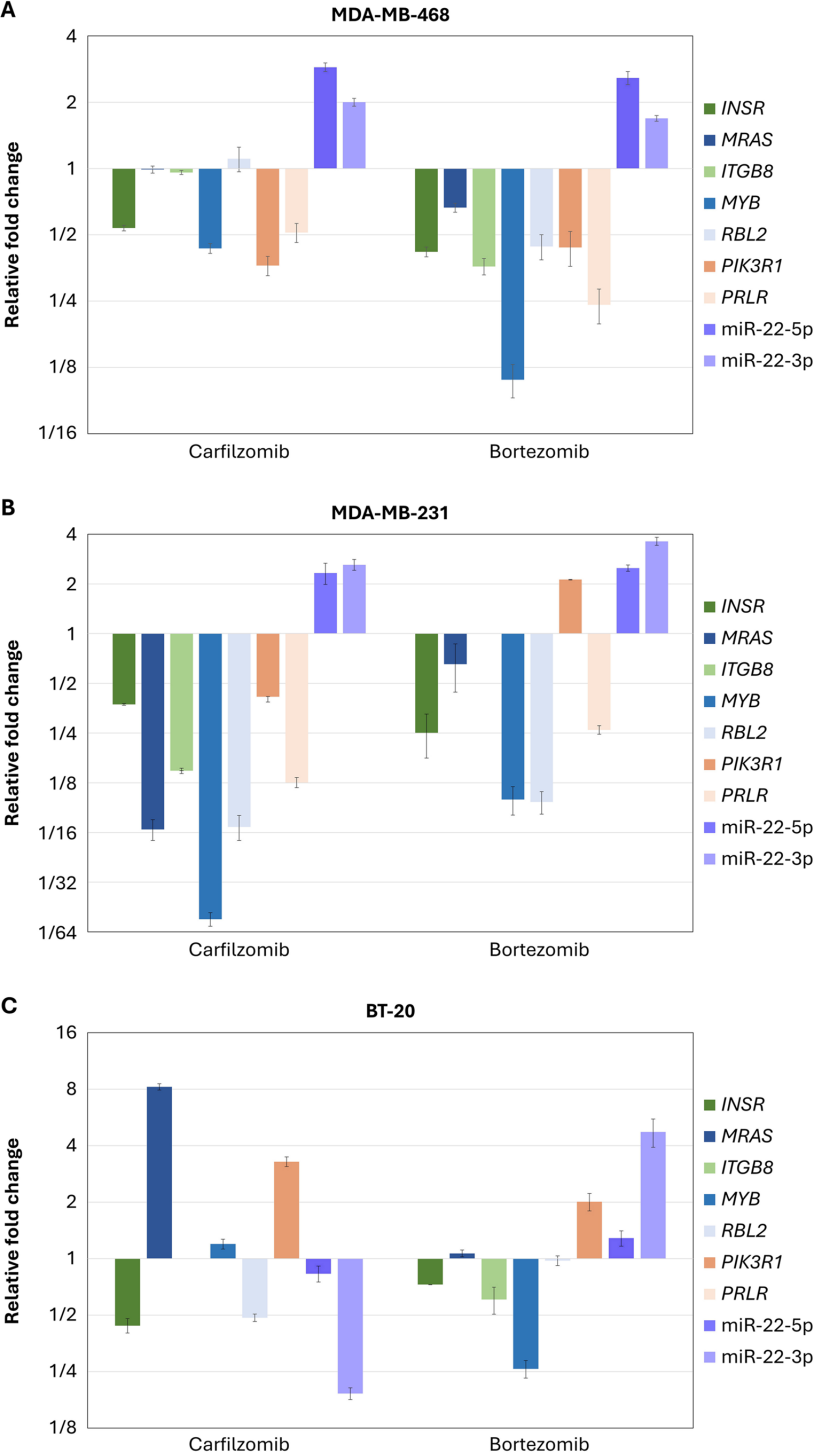
Levels of selected miRNAs and mRNA targets at 24 h post-treatment with inhibitors in the MDA-MB-468 **(A)**, MDA-MB-231 **(B)**, and BT-20 **(C)** cell lines

Changes in the expression of selected miRNAs and mRNAs that were observed in the MDA-MB-468 cell line were evaluated in two more TNBC cancer cell lines. In the TNBC cell lines (Fig. 6), the levels of miR-22-5p and miR-22-3p appeared to increase after the effect with an inhibitor in two and three cell lines, respectively. Furthermore, the levels of selected mRNA targets appear to be reduced, with the expression profile being similar in the cell lines of metastatic origin, MDA-MB-468, and MDA-MB-231.

### Regulation of PI3K/AKT pathway components by mir-22 overexpression

**Fig. 7:**
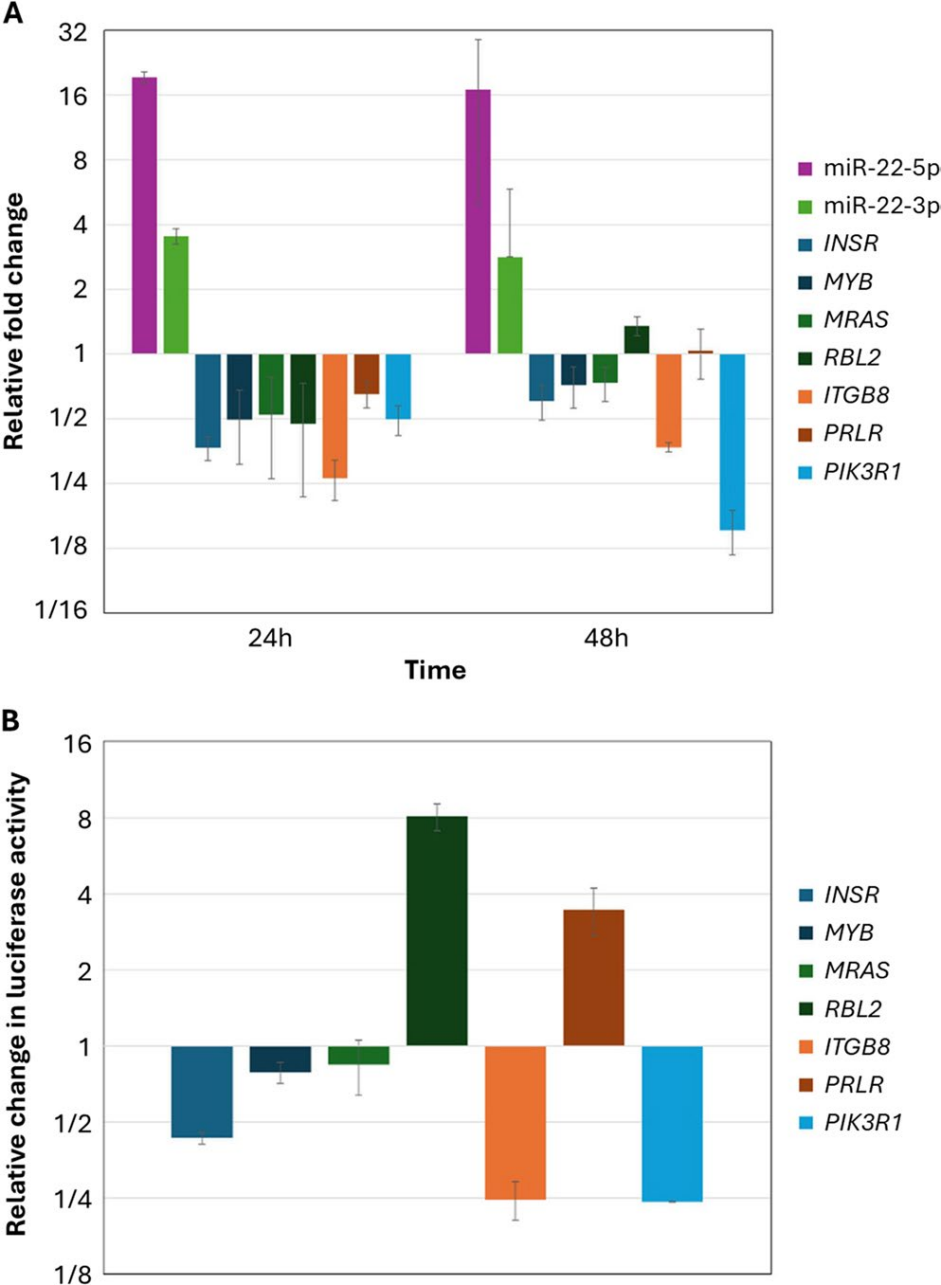
Relative alterations in the levels of miR-22-5p, miR-22-3p, and their targets, following the overexpression of mir-22 in the MDA-MB-468 cell line. The fold change of their levels **(A)**, and the change in luciferase activity after the co-transfection of cells with mir-22 and the mRNA 3´-UTR overexpression plasmids **(B)** are depicted

The transfection of the MDA-MB-468 cell line with pCMV6-Neo-mir-22 resulted in a significant upregulation of miR-22-5p levels, with a relevant downregulation of its predicted targets *RBL2*, *MYB*, *ITGB8*, *INSR*, *PIK3R1*, and a slight decrease of *PRLR* (Fig. 7A). Aiming to validate the regulatory interaction with its targets we performed a luciferase activity assay by co-transfecting the MDA-MB-468 cell line with pCMV6-Neo-mir-22 and the psiCHECK-2-3´UTR of each of the predicted targets. The luciferase assay supported a direct interaction between miR-22-5p and *INSR*, *ITGB8*, and *PIK3R1* as a reduction of the luciferase activity was observed (Fig. 7B). These three genes which appear altered by miR-22-5p are significant components of the PI3K/AKT signaling pathway.

Regarding miR-22-3p, its expression seems to increase after transfection of cells with pCMV6-Neo-mir-22, while that of its target *MRAS* is reduced. However, in the investigation of the functional interaction with the luciferase assay, the interaction did not seem to be confirmed.

### Effect of mir-22 overexpression on MDA-MB-468 TNBC cell proliferation and migration

**Fig. 8:**
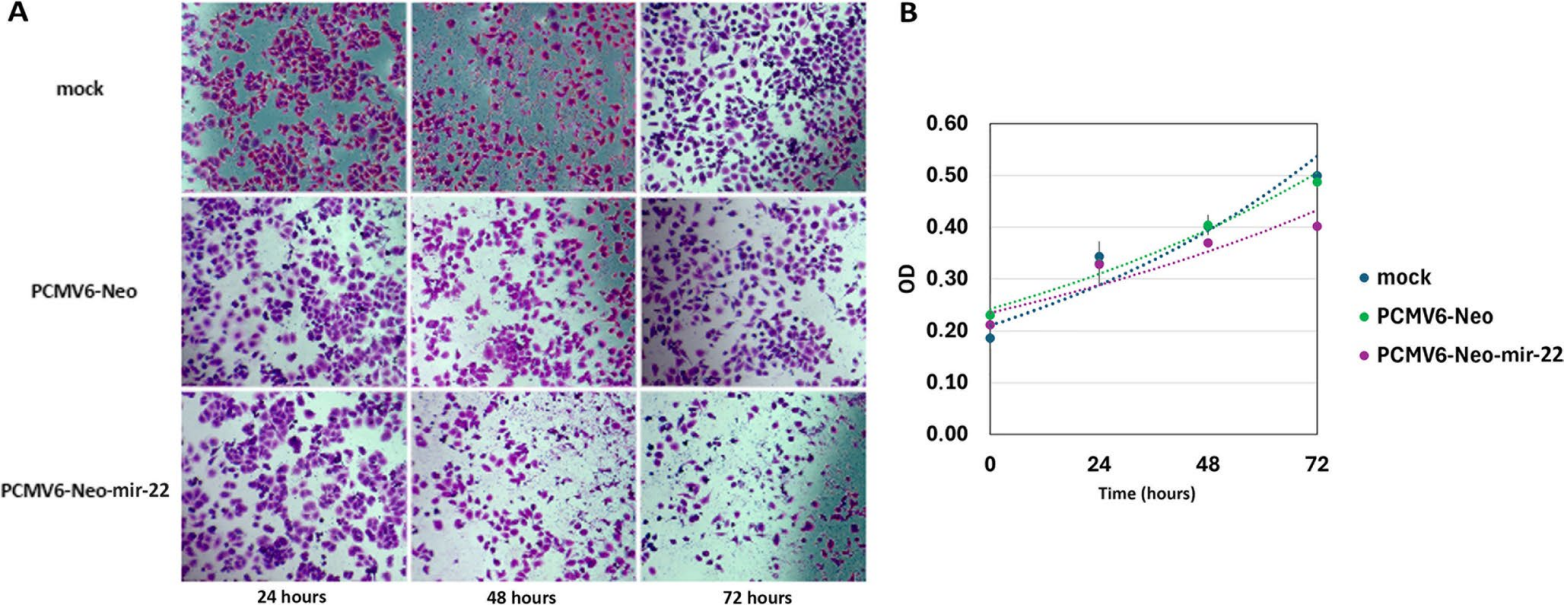
Pictures of cell culture (**A**) and proliferation curves (**B**) of the MDA-MB-468 cell line after treatment with the transfection reagents (mock), the empty pCMV6-Neo vector or the pCMV6-Neo-mir-22 vector

The proliferative and migratory capacity of cells was assessed, after the overexpression of mir-22 in the MDA-MB-468 cell line. This overexpression resulted in a decreased proliferative capacity of the cell culture (Fig. 8). Moreover, a reduction in the migratory potential of the MDA-MB-468 cells was also observed after the upregulation of mir-22 (Fig. S1).

## Discussion

Cell signaling is majorly deregulated in cancerous conditions, and the deregulation of these pathways results in characteristic changes contributing to BrCa pathogenesis and disease progression. Numerous genes have been implicated in the disruption of cell signaling pathways, and growing evidence indicates that many are post-transcriptionally regulated by sncRNAs through the RNA interference mechanism. In our study, a combinational sequencing approach was performed for the identification of novel post-transcriptional regulatory interactions influencing BrCa cell signaling, following treatment with PIs. Pursuing a broad analysis of BrCa gene expression, cell lines deriving from the major molecular subtypes were incorporated. Moreover, an integration of results deriving from small RNA sequencing and poly(A)-RNA sequencing was performed, in an effort to identify specific sncRNA-mRNA interactions. The effect of PIs at a mRNA-level appears most significant in the Luminal B and TNBC cell lines, as more genes appeared with significantly altered expression in comparison to the other molecular subtypes. Moreover, an alteration in significant pathways that are related to BrCa, including pathways in cancer, the MAPK and PI3K/AKT signaling pathways, cell cycle and protein related processes such as the function of the proteasome, lysosome, and ribosome were also observed. These observations are in accordance with previous information, suggesting that PIs influence numerous significant signaling pathways in BrCa (Katsaraki et al. 2024).

In the next step, we focused only on genes that are part of the MAPK, PI3K/AKT, and NFκB signaling pathways, with most genes contributing to cell signaling and with altered expression being observed in the MDA-MB-468 cell line. Interestingly, *EGF*,* GADD45A*,* GADD45G*,* HSPA1B*, and *SPP1* appeared significantly upregulated in seven conditions studied, after treatment with PIs. The coordinated increase of these genes suggests an alteration of adaptive survival and proliferative programs following PIs treatment universally in all cell lines. In BrCa based on bibliography, EGF signaling regulates proliferation and invasion, GADD45 family members modulate DNA damage responses and tumor formation, *HSPA1B* levels have been found altered under specific conditions, and elevated *SPP1* is associated with poor prognosis and recurrence (Masuda et al. 2012; Pietrasik et al. 2020; Wang et al. 2008, 2018; Xiong et al. 2023; Xu et al. 2023; Zhang et al. 2021) (Gothlin Eremo et al. 2020).

Furthermore, *CXCL2*, *HSPA6*, and *LY96* appeared upregulated in the two luminal and the TNBC cell lines after treatment with both the PIs, whereas *FGFR3*, and *MAP3K12* appeared downregulated, and *PPP2R2C* upregulated in the BT-474 and MDA-MB-468 cell lines. Based on previously published data, *CXCL2* may have a dual role in BrCa, while *HSPA6* functions as a tumor suppressor (Shen et al. 2021). Moreover, targeting *FGFR3* has been proposed as a therapeutic strategy in TNBC (Chew et al. 2020). Further focusing on the results of our study and besides the overexpression of genes in the majority of the cases that were studied, *CDKN1A*, *INSR*, *MRAS*, *PIK3R1*, and *PRLR* displayed altered expression uniquely in the TNBC MDA-MB-468 cell line. The products of these genes that regulate cell cycle progression as well as cell signaling pathways according to literature prompted further mechanistic investigation in this subtype.

Beyond coding genes, our integrative analysis uncovered substantial remodeling of the sncRNA landscape. Several piRNAs such as piR-36,318, piR-39,245, and piR-36,036 and tRNA-derived RNA fragments such as i-tRF-TRQ-CTG3(1), 5’-tRF-TRV-CAC14, and 5’-tRF-NMTRL-TAA1 exhibited marked and subtype-specific alterations. On the contrary 5’-tRF-TRN-GTT11, and 3’-tRF-TRQ-CTG3 appeared significantly downregulated in all the combinations studied. Collectively, the most affected tRNA-derived RNA fragments are predicted to alter major oncogenic pathways including MAPK, PI3K/AKT, and NFκB and important BrCa related genes, indicating based on literature the modulation of essential processes such as genomic stability, cell cycle progression, proliferation, inflammation, metastasis and angiogenesis (Al Kawas et al. 2022; Herschkowitz et al. 2008; Mitchell et al. 2025; Stipp and Acco 2025).

Aiming to identify miRNAs with a significant alteration in their expression following treatment only three miRNAs, namely miR-489-3p, miR-876-3, and miR-1827 appeared with significantly altered levels in all eight conditions studied. Other miRNAs, such as miR-2276-5p and miR-320e appeared altered in a more subtype specific manner. Only a limited subset demonstrated consistent dysregulation across experimental conditions, highlighting selective rather than global miRNA reprogramming. Significant pathways, including those involved in BrCa, apoptosis, p53 signaling, and the MAPK and PI3K/AKT cascades, appear to be influenced from gene set enrichment analysis, supporting a substantial predicted impact of the altered miRNAs on cell signaling networks. Moreover, key biological processes such as regulation of gene expression, control of translation, cell-cycle regulation, and proteasome-mediated protein degradation also seem to be affected. According to literature, the assessment of the regulatory potential of the aforementioned miRNAs in BrCa may reveal important results as they have been characterized with contradictory roles in BrCa or as prognostic markers in other malignancies (Perez-Carbonell et al. 2015; Sun et al. 2021).

Following the broad elucidation of gene expression covering the regulator (sncRNA) and the regulated molecule (mRNA), the integration of the results unraveled potential interactions that were selected for further assessment. The analysis was focused on predicted interactions where a miRNA is upregulated, and potential mRNA targets are downregulated after treatment. In addition, already confirmed interactions in BrCa, interactions in other cancer types, and potential interactions that were worth further studying were found. Interestingly, miR-22 emerged as a strong candidate regulator in TNBC models, as integrative analysis revealed that several predicted miR-22 targets were concurrently downregulated in the MDA-MB-468 cell line following PI treatment, supporting a functional regulatory relationship.

Validation experiments across three TNBC cell lines confirmed increased levels of miR-22-5p and miR-22-3p after inhibitor treatment. Furthermore, the levels of selected mRNA targets appeared to be reduced with the expression profile being similar in the cell lines of metastatic origin, MDA-MB-468, and MDA-MB-231. The overexpression of mir-22 in the TNBC MDA-MB-468 cell line resulted in a significant upregulation of miR-22-5p levels, with a relevant downregulation of its predicted targets *RBL2*, *MYB*, *ITGB8*, *INSR*, *PIK3R1*, and a slight decrease of *PRLR*. The direct interaction of miR-22-5p with *ITGB8*, *INSR*, and *PIK3R1* was validated using the luciferase activity assay. These three genes which appear altered by miR-22-5p are significant components of the PI3K/AKT signaling pathway. Furthermore, the overexpression of miR-22 resulted in an inhibition of the proliferative and migratory capacity of cells.

These findings suggest that miR-22-5p may act as a modulator of PI3K/AKT signaling in TNBC, affecting cell signaling and the relative cellular properties. This finding is particularly relevant, as according to published data the PI3K/AKT pathway is frequently altered in TNBC (Cancer Genome Atlas 2012; Ortega et al. 2020). Integrin subunit beta 8 (ITGB8), a member of the integrin family involved in cell–matrix adhesion and signaling, has been reported to be upregulated in ovarian cancer and linked to invasion. Moreover, it has been associated with lung metastasis in BrCa (Culhane and Quackenbush 2009; Hood and Cheresh 2002; Peng et al. 2020). According to previous studies, the insulin receptor (INSR), a receptor tyrosine kinase, promotes BrCa tumor initiation via MAPK, PI3K/AKT, and JAK/STAT signaling, while its deletion inhibits tumorigenesis. A circular RNA from *INSR* also regulates apoptosis and metabolism in cardiomyocytes, protecting against doxorubicin-induced death. (Lu et al. 2022; Podmore et al. 2023). As described in the literature, the phosphoinositide-3-kinase regulatory subunit 1 (PIK3R1) which is the regulatory subunit of PI3K, controls cell proliferation and survival. In BrCa, it may act as a tumor suppressor, targeted by miR-21 and miR-155, with its downregulation being linked to poorer progression free survival and frequent mutations in BrCa (Cizkova et al. 2013; Cobleigh et al. 2024; Kim et al. 2018; Yan et al. 2016). The simultaneous targeting of multiple pathway components by miR-22-5p may indicate coordinated modulation of PI3K/AKT signaling rather than isolated gene suppression. Importantly, the observed reduction in proliferation and migration following miR-22 overexpression supports a predominantly antitumor role in the TNBC cellular context examined here. These results underscore the context-dependent nature of miRNA function and highlight the importance of assessing regulatory effects at the network level.

Besides our effort that resulted in the identification of one regulatory molecule with indications for its ability to alter the levels of three key components of an important cell signaling pathway, additional dysregulated sncRNAs including specific tRNA-derived RNA fragments and piRNAs are predicted to influence key BrCa associated pathways. Their subtype specific expression patterns suggest potential significant roles, but further mechanistic studies are required to define their direct targets and biological impact. Moreover, experiments were conducted in established BrCa cell lines, which may not fully reflect tumor heterogeneity or microenvironmental influences observed in patients. Additionally, pathway modulation was estimated from transcriptomic enrichment and target expression changes, without direct measurement of downstream signaling activity such as phosphorylation of PI3K/AKT or MAPK components. Finally, in-vivo validation and analysis of patient-derived samples were beyond the scope of the present study. Therefore, further studies are required to determine the clinical and biological relevance of the identified regulatory axes.

A notable point that appears essential to assess in every context is the bioavailability of sncRNAs that appear as significant regulators. Apart from the stability of the RNA itself, molecules such as long non-coding RNAs and circular RNAs may significantly alter the regulatory effect of sncRNAs, by acting as competing endogenous RNAs (Papatsirou et al. 2020; Yang et al. 2023). Therefore, the time-dependent regulatory potential of these molecules should also be assessed. The incorporation of experimentally verified as well as predicted interactions between sncRNAs and their respective targets into a specific database appears crucial, as it enables the scientific community to extract key regulatory relationships. Furthermore, even though the validated regulatory interactions of sncRNAs and mRNA targets are augmenting, more high-throughput experiments such as AGO CLIP-sequencing appear essential for the assessment of regulatory interactions in a large-scale strategy. Lastly, the implementation of a relative combinational sequencing approach could also be implicated in studies incorporating samples of patients at the time of diagnosis, post-treatment, as well as after recurrence. This approach would allow the identification of a limited number of specific sncRNA-mRNA or lncRNA/circRNA-sncRNA-mRNA regulatory axes with an important effect in BrCa. Furthermore, the identification of specific sncRNA molecules with a stable expression during each of the abovementioned stages appears crucial for the proposal of molecules with therapeutic utility. These findings could contribute to the identification of biomarkers and the development of targeted therapies, addressing unmet needs in BrCa patients’ management.

Together, our findings indicate both shared and subtype-specific transcriptomic alterations in BrCa cell line models following PI treatment that impacts coding RNAs as well as a variety of sncRNAs, such as miRNAs, tRNA-derived RNA fragments, and piRNAs. The integrative sequencing analysis unraveled predicted regulatory sncRNA-mRNA axes potentially altering important oncogenic signaling pathways, such as PI3K/AKT and MAPK and other cellular processes. The miR-22 was found to be continuously elevated in TNBC models. Functional tests showed that miR-22-5p’s overexpression inhibits TNBC cell proliferation and migration, while experimental validation verified its direct interaction with *INSR*, *ITGB8*, and *PIK3R1*, which are essential elements of the PI3K/AKT pathway.

Apart from this miRNA, other sncRNAs, such as a number of tRNA-derived RNA fragments and piRNAs, showed notable subtype-specific changes and are expected to target genes related to survival, proliferation, metastasis, and angiogenesis. These findings suggest that several sncRNA classes interact together to modify signaling and transcriptomic networks. Collectively, our findings highlight the contribution of sncRNAs to the regulation of key signaling networks in BrCa and provide a framework that may aid in the identification of regulatory axes, supporting the discovery of novel biomarkers and therapeutic targets.

## Supplementary Information

Below is the link to the electronic supplementary material.

Supplementary Material 1

Supplementary Material 2

Supplementary Material 3

Supplementary Material 4

Supplementary Material 5

Supplementary Material 6

Supplementary Material 7

Supplementary Material 8

## Data Availability

The raw nanopore sequencing reads have been deposited to the Sequence Read Archive (SRA) of NCBI, with BioProject accession numbers PRJNA1415803 (small RNA-seq data) and PRJNA1415864 (polyA RNA-seq data). All other data will be made available upon request.
