# Supplementary material for: Unraveling small non-coding RNAs with a significant post-transcriptional impact on breast cancer cell signaling, using a combinational sequencing approach
Source: Funct Integr Genomics. 2026 Mar 23;26(1):73. doi: 10.1007/s10142-026-01856-6 (PMC13006467; doi:10.1007/s10142-026-01856-6)
Supplement: Supplementary file 1 — Supplementary Material 1 [file 10142_2026_1856_MOESM1_ESM.docx]

**Fig. S1** Pictures of the wound healing assay **(A)** and quantification of the reduction in migratory capacity **(B)** at 48, 72, and 96 hours following treatment of the MDA-MB-468 cell line with the transfection reagents (mock), the empty pCMV6-Neo vector, and the pCMV6-Neo-mir-22 vector.
