# Supplementary material for: Unraveling small non-coding RNAs with a significant post-transcriptional impact on breast cancer cell signaling, using a combinational sequencing approach
Source: Funct Integr Genomics. 2026 Mar 23;26(1):73. doi: 10.1007/s10142-026-01856-6 (PMC13006467; doi:10.1007/s10142-026-01856-6)
Supplement: Supplementary file 2 — Supplementary Material 2 [file 10142_2026_1856_MOESM2_ESM.docx]

**Table S1** Characteristics of primers used for the amplification of selected miRNAs, mRNAs, as well as regions in the multiple cloning sites of plasmids.

| **Target** | **Direction** | **Sequence (5΄→ 3΄)** | **Length (nucleotides)** |
| --- | --- | --- | --- |
| *SNORD43* | Sense | ACTTATTGACGGGCGGACA | 19 |
| *SNORD44* | Sense | AGCAAATGCTGACTGAACATGA | 22 |
| hsa-miR-22-5p | Sense | CGAGTTCTTCAGTGGCAAGCTTTAA | 25 |
| hsa-miR-22-3p | Sense | AAGCTGCCAGTTGAAGAACTGTAA | 24 |
| (Universal primer) | Antisense | GCGAGCACAGAATTAATACGAC | 22 |
| *GAPDH* | Sense | ATGGGGAAGGTGAAGGTCG | 19 |
|  | Antisense | TGACAAGCTTCCCGTTCTCA | 20 |
| *INSR* | Sense | CACTGCCAGAAAGTTTGCCC | 20 |
|  | Antisense | TGCCGTCCAGGTAGAAGTTG | 20 |
| *ITGB8* | Sense | CTGGGCCAAGGTGAAGACAA | 20 |
|  | Antisense | TCAACTGAGCAGCCTTTGCT | 20 |
| *MRAS* | Sense | GCGTCAAAGACAGGGAGTCA | 20 |
|  | Antisense | CGACATTGAGAGGTGGGTCC | 20 |
| *MYB* | Sense | GAAGACCCCGGCACAGCATA | 20 |
|  | Antisense | GACGCTTTCCAGACTTGGGA | 20 |
| *PIK3R1* | Sense | ATGCCTGCTCTGTAGTGGTG | 20 |
|  | Antisense | CTGCACAAGGGAGGTGTGTT | 20 |
| *PRLR* | Sense | TGGTCTCCACCTACCCTGAT | 20 |
|  | Antisense | CCCAGCAAAATGGATCTCCCA | 21 |
| *RBL2* | Sense | GCTACACGCTGGAGGGAAAT | 20 |
|  | Antisense | TCCTTCCACTGTCCCTTTGC | 20 |
| *MIR22* | Sense | TTTATAGAGCTCGTCTTGCTGCTCAGCGAGGTTAA | 35 |
|  | Antisense | TTTATGGAATTCCTCTACTCCTCAATCCAGCCA | 33 |
| psiCHECK2 MCS^1^ | Sense | GGAGGACGCTCCAGATGAAA | 20 |
|  | Antisense | ACCAACACACAGATGTAATGAAAA | 24 |
| PCMV6-Neo MCS^1^ | Sense | CAACGGGACTTTCCAAAATGTCG | 23 |
|  | Antisense | ATTAGGACAAGGCTGGTGGG | 20 |

^1^Multi-cloning site.
