# Supplementary material for: Unraveling small non-coding RNAs with a significant post-transcriptional impact on breast cancer cell signaling, using a combinational sequencing approach
Source: Funct Integr Genomics. 2026 Mar 23;26(1):73. doi: 10.1007/s10142-026-01856-6 (PMC13006467; doi:10.1007/s10142-026-01856-6)
Supplement: Supplementary file 4 — Supplementary Material 4 [file 10142_2026_1856_MOESM4_ESM.docx]

**Table S****3** Sequences of tRNA-derived RNA fragments exhibiting the highest alterations in their expression levels.

| **tRNA-derived RNA fragment** | **Sequence (5΄→ 3΄)** |
| --- | --- |
| 3'-tRF-TRQ-CTG3 | CGAGTTCGAGTCTCGGTGGAACCTCCA |
| 5'-tRF-NMTRL-TAA1 | GTTAAGATGGCAGAGCCTGGTAAT |
| 5'-tRF-TRG-CCC4 | GCGTTGGTGGTTTAGTGGTAGAATTCTCGCC |
| 5'-tRF-TRN-ATT1 | GTCTCTGTGGCGCAATCGGTC |
| 5'-tRF-TRN-GTT11 | GTCTCTGTGGTGCAATCGG |
| 5'-tRF-TRN-GTT8 | GTCTCTGTGGCGCAATCGGC |
| 5'-tRF-TRV-CAC14 | GCTTCTGTAATGTAGTGGTTATC |
| i-tRF-TRD-GTC4(2) | CTGCCTGTCACGCGGGAGAC |
| i-tRF-TRE-CTC12 | TGTGGTTAGGATTCGGCGCTC |
| i-tRF-TRI-TAT3(1) | AATGCCGAGGTTGTGAGTTCAAGC |
| i-tRF-TRK-TTT14(1) | ATCTGAGGGTCCAGGGTTCATGTCCCTGTT |
| i-tRF-TRL-AAG2(3) | CTCTTCGGGGGCGTGGGTTCGAA |
| i-tRF-TRL-CAG1(5) | CGTTCAGGTCGCAGTCTCCCCTGG |
| i-tRF-TRQ-CTG3(1) | AATGGTGAGCACTCTGGACTCTGAATCCAG |
| i-tRF-TRQ-CTG3(2) | ATGGTGAGCACTCTGGACTCTGAATCCAGC |
| i-tRF-TRY-GTA1(9) | GTTGGTAGAGCGGAGGACTGTAGATCC |
| Leader-NMTRL-TAA3-1 | ATTATCTCAATCTAACAAATCATCACACACCCTACCCAAGAACAGGGTTT |
| Leader-NMTRQ-TTG1-1 | CCAACATTTTCGGAGTATGGGCCCGATAGCTTATTTAGCTGACCTTACT |
| Trailer-TRG-GCC4-1 | GCACGCCCTCCCATTTTGGTGCTGCAGCAGCACCAAGGCGTAGCTGCGTT |
| Trailer-TRQ-CTG3-1 | TTCTGTTTAATTAGGACGGCAATGTTGTGTTTTACTCCCTAAATGGAATG |
