# Supplementary material for: Unraveling small non-coding RNAs with a significant post-transcriptional impact on breast cancer cell signaling, using a combinational sequencing approach
Source: Funct Integr Genomics. 2026 Mar 23;26(1):73. doi: 10.1007/s10142-026-01856-6 (PMC13006467; doi:10.1007/s10142-026-01856-6)
Supplement: Supplementary file 7 — Supplementary Material 7 [file 10142_2026_1856_MOESM7_ESM.docx]

**Description of Supplementary file**

FASTA sequences of 3'-untranslated regions (3'-UTRs) that were recombined with the expression vector psiCHECK-2: Supplementary File.fasta
